# Non-Invasive and Label-Free On-Chip Impedance Monitoring of Heatstroke

**DOI:** 10.3390/bios13070686

**Published:** 2023-06-27

**Authors:** Yueli Zhao, Weihua Fan, Anwei Liu, Shihua Pan, Chongxiao Xu, Hailun Peng, Bingling Yin, Xiaodong Wang, Jianhua Dong, Zhiguo Pan

**Affiliations:** 1 Guangzhou University of Chinese Medicine, Guangzhou 510006, China; 20201111187@stu.gzucm.edu.cn (Y.Z.); 20221111561@stu.gzucm.edu.cn (B.Y.); 2 Guangzhou Institute of Biomedicine and Health, Chinese Academy of Sciences, Guangzhou 510530, China; fan_weihua@gibh.ac.cn (W.F.); 2020219451@stu.gzhmu.edu.cn (S.P.); dong_jianhua@gibh.ac.cn (J.D.); 3 Department of Intensive Care Unit, General Hospital of Southern Theatre Command of PLA, Guangzhou 510010, China; anweiliu2014@163.com (A.L.); fanteny@tom.com (C.X.); 17825846221@163.com (H.P.); 4 Department of Biochemistry and Molecular Biology, GMU-GIBH Joint School of Life Sciences, Medical University, Guangzhou 511436, China; 5 Southern Medical University, Guangzhou 510515, China; 6 Engineering Research Center for Semiconductor Integrated Technology, Institute of Semiconductors, Chinese Academy of Sciences, Beijing 100083, China; xdwang@semi.ac.cn

**Keywords:** heatstroke, biochip, impedance

## Abstract

Heatstroke (HS) is a life-threatening injury requiring neurocritical care which could lead to central nervous system dysfunction and severe multiple organ failure syndrome. The cell–cell adhesion and cell permeability are two key factors for characterizing HS. To investigate the process of HS, a biochip-based electrical model was proposed and applied to HS. During the process, the value of TEER is associated with cell permeability and C_I_ which represents cell–cell adhesion decreases that are consistent with the reduction in cell–cell adhesion and cell permeability characterized by proteins (occludin, VE-Cadherin and ZO-1) and RNA level. The results imply that the model can be used to monitor the biological process and other biomedical applications.

## 1. Introduction

Heatstroke is a life-threatening illness characterized by an elevated core temperature above 40 °C and central nervous system dysfunction [1]. It results from the imbalance between heat production and dissipation within the body. Recent studies have revealed death rates as high as 10–15% in general heatstroke cases and up to 40% in severe cases that suffer from disseminated intravascular coagulation (DIC), acute kidney injury (AKI), rhabdomyolysis (RM), and even multiple organ dysfunction syndrome (MODS) [2]. Current consensus attributes the immediate response to heat stress to the injury of vascular endothelial cells (VECs), which triggers subsequent coagulation activation and inflammation, both of which crucially contribute to the progression of heatstroke. Despite this, inadequate research has hampered the assessment of VEC damage, thus stalling the development of disease evaluation and effective treatment for heatstroke [3,4,5,6].

The current methods for labeling VEC damage have several limitations, such as invasiveness, complexity, and imprecision [7,8]. For instance, the Transwell experiment, a traditional method frequently used in cell biology research, requires fixing cells with 4% paraformaldehyde prior to the subsequent step, which may damage cell vitality. Furthermore, trans epithelial electrical resistance (TEER), commonly adopted to quantify cell permeability in a non-invasive and label-free manner, can only be applied to monolayer cells, not individual cells [9,10]. However, due to recent advances in microfluidic technology, nanofabrication, and integrated sensors, biochips have emerged as a reliable alternative. Biochips can precisely simulate the in vivo physical environment, thanks to their ability to control the flow of culture medium into cell chambers [11]. Consequently, the traditional cell culture systems utilizing culture flasks and plates has gradually been abandoned.

Endothelial cells form a semi-selective barrier in the body that separates blood from organs and tissues, playing a crucial role in maintaining the overall stability of the body. Due to cell–cell interactions, the endothelial cells adjacent to each other form tight junctions (TJs), adhesion junctions (AJs), and gap junctions (GJs) [12,13]. The molecular composition of tight junction proteins (TJs) includes: claudin, occludin, junctional adhesion molecules (JAMs), and zonula occludens (ZOs) [14,15,16,17,18,19,20,21]; the “basal” tissue of adhesion junctions is provided by vascular endothelial cadherin (VE-cadherin). By decreasing the expression of the tight junction structural proteins ZO-1 and occludin in vascular endothelial cells, heatstroke prevents endothelial cells from forming tight junction complexes, resulting in damage to the endothelial cell barrier structure, thereby increasing vascular permeability [2].

**Figure 1:**
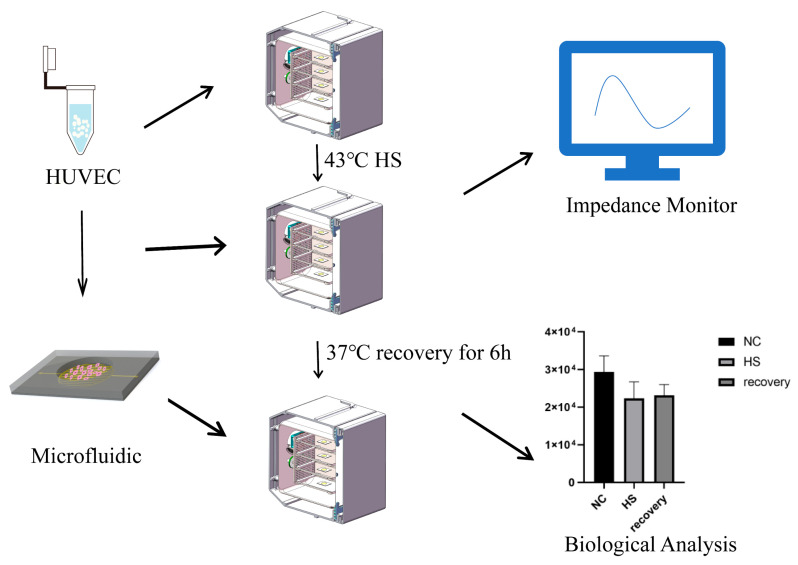
Flowchart of this study: First, the HUVECs were cultured on a biochip. Second, a heat stroke model was constructed. Finally, different samples were collected for analysis and the impedance of the heat-stressed HUVECs was measured by an instrument.

In the present study, we proposed a novel biochip to test the electrical changes of cells under heat stress. The new biochip could be used to monitor TEER and cell–cell integrin capacitance in real time when VECs were exposed to varying levels of heat stress condition. What is more, we also administrated that the electrical characteristics were in agreement with the change of specific proteins and RNA expression reflecting cell permeability. This study provides a novel method to continuously monitor VECs damage under heat stress, which is helpful for clinicians to understand the patient’s vascular endothelial cell damage and inflammation. The overall research thought is shown in Figure 1.

## 2. Materials and Methods

### 2.1. Design and Fabrication of the Chip

**Figure 2:**
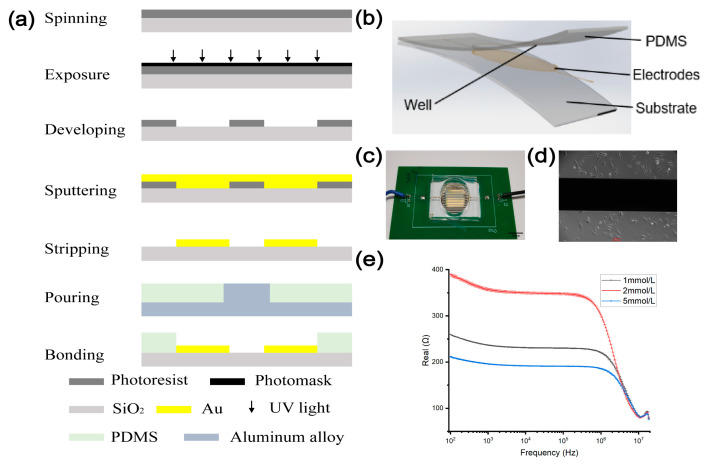
(**a**) Fabrication process of the PDMS. On the silicon dioxide after photoetching, it is exposed by UV light. After the photoresist was removed, the mold can be used. The PDMS mix is then poured and demolded to obtain the PDMS layer. Finally, the electrodes were bonded together with PDMS to form a complete structure. (**b**) 3D rendering of the chip architecture. (**c**) Images of the chip. (**d**) The microscope view of cells on the chip. (**e**) The sensitivity of the biochip was tested using different concentrations (1/2/5 mmol/L) of glucose.

The device is shown in Figure 2a, and comprises a substrate layer, an electrode layer and a construction layer. The fabrication process of the device comprises the following key steps: First, Au/Ti electrodes were manufactured by a lift-off process. Next, the construction layers were manufactured by pouring the PDMS and curing agent (ratio of 10:1) on an aluminum alloy mold. After curing 30 min with 60 °C and peeling from the mold, the finished device was prepared by aligning and bonding the construction layer to the electrode chip (Figure 2b).

### 2.2. Cell Preparation (Human Umbilical Vein Endothelial Cells Culture)

Human umbilical vein endothelial cells (HUVECs) were purchased from Procell Co., Ltd., Santa Ana, CA, USA) The experiments were conducted using cells from passage 4 to 7, all of which were grown in 10 cm culture dishes (Greiner Bio-One, Kremsmünster, Austria). The cells were then subcultured every 3 to 5 days, and the culture medium (Procell) was changed every 2 days. The cells were subcultured at 90% confluence, then we use 2 mL of 0.25% trypsin-EDTA (Gibco, Billings, MT, USA) to digest cells. A new culture flask was prepared, and the remaining cells were used to observe the prepared microfluidic and 6-well plate (Greiner Bio-One) that had been coated with 100 μg/mL of fibronectin (ThermoFisher Scientific, Waltham, MA, USA) for 2 to 4 h at 37 °C. The control cells were maintained in an incubator at 37 °C. For heat stress induction, cells were subjected to 43 °C for 2 h and then to 37 °C for 6 h. In addition, the cell incubator used in this study was developed by our research group, and the temperature fluctuation was ±0.2 °C.

### 2.3. Western Blotting

First, protein samples from cells were prepared using radioimmunoprecipitation assay (RIPA) lysis buffer. The protein samples were then subjected to sodium dodecyl sulfate-polyacrylamide gel electrophoresis (SDS-PAGE) followed by electroblotting onto polyvinylidene fluoride (PVDF) membranes. The PVDF membrane was connected to the positive electrode on one side (red) and the gel to the negative electrode on the other side (black). The membranes were then probed with monoclonal antibodies against ZO-1 (Proteintech group), polyclonal antibodies against VE-cadherin (Cell Signaling Technology), occludin (Proteintech), and GAPDH (GeneTex). Finally, the protein bands were visualized using chemiluminescence detection reagents (Advansta), and densitometric analysis was conducted using imaging processing software (Image J 1.8.0_172).

### 2.4. Quantitative Real-Time Polymerase Chain Reaction (qRT-PCR)

Total RNA was extracted with TRIzol^®^ reagent (Invitrogen, Waltham, MA, USA) and cDNA was prepared using HiScript^®^ III RT SuperMix for qPCR (+gDNA wiper) (Vazyme, Nanjing, China) according to the manufacturer’s protocol. Quantitative RT-PCR of heat-stressed HUVECs was performed using SYBR^®^ Green (Bio-Rad) and the following primers are shown at the 5′ to 3′ ends’ (Table 1). cDNA was obtained by reverse transcription at 1:20 dilution, and the diluted cDNA was used as a template. The reaction system for each well is formulated in the following proportions (Table 2). The data were normalized to GAPDH expression, and relative expression changes were analyzed using the ΔCt method. Instrument software sets the fluorescence value of the 3rd–15th cycles as the baseline. The threshold is 10 times the standard deviation of the baseline. The Ct value of the template has a linear relationship with the logarithm of the initial copy number of the template. The higher the initial template concentration, the smaller the Ct value, and the lower the initial template concentration, the larger the Ct value. When the PCR cycle reaches the cycle at which the Ct value is located, it just enters the true exponential amplification period (logarithmic phase). At this time, the small error has not been magnified, and the reproducibility of the Ct value is good, that is, for the same amount of initial template, the obtained Ct value is relatively stable.

### 2.5. Chip Circuit Model

**Figure 3:**
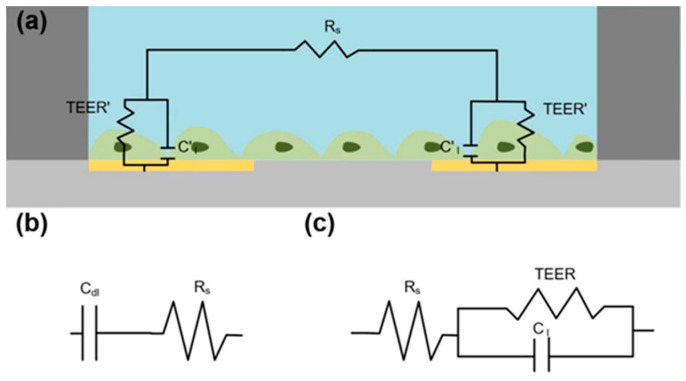
The diagram of the equivalent circuit model of microfluidics: (**a**) Model of the system and corresponding equivalent electric model with (**b**) no cells and (**c**) a cell monolayer Rs, C**_dl_** and C_I_ represent the resistance of the medium, the capacitance of the double layer between the electrodes and the medium, and capacitance of cell–cell adhesion.

According to the physical properties of the subjects, an equivalent circuit model is designed for measuring the properties of monolayer cells using our system. The system and the corresponding equivalent circuit model are shown in Figure 3a, and the corresponding simplified circuit is shown in Figure 3b,c. The capacitance, CI, which presents the cell–cell integrin, can be calculated using a simplified circuit from Figure 3 and Equation (2). The TEER, which is associated with the cell body, is calculated using Equation (3) which is described in detail in the Supplementary Materials.

### 2.6. Trans-Epithelial Electrical Resistance (TEER) and Capacitance of Cell-Cell Adhesion Measurement

The biochip was glued onto a custom-made printed circuit board (PCB). The electrodes were connected to the PCB through a copper wire, and the PCB was connected to an impedance analyzer (HF2LI, Zurich Instrument, Zurich, Switzerland). The calibration (blank medium) served as a baseline, and the cell impedance was measured using the HF2LI instrument with an input of 100 mV. The frequency range was from 100 Hz to 1 MHz, and 10 points were measured per decade. First the HUVECs in the control group were cultured at 37 °C in 5% CO_2_ until the cells occupied approximately 80–90% of the well area of the biochip, and the impedance was then measured. The biochip in the experimental group was incubated for 2 h in the 43 °C, 5% CO_2_ incubator, and the impedance was recorded. Finally, the biochip in the recovery group was incubated for 6 h in an incubator at 37 °C in 5% CO_2_, and the impedance was recorded. Images were obtained using a fluorescence microscope (IX73, Nikon, Tokyo, Japan).

### 2.7. Immunofluorescence Assay

The culture medium was removed and washed three times with PBS. The cells were fixed with 4% PFA (LEAGENE) at room temperature for 15 min, which was removed from the refrigerator and stored at room temperature beforehand. Washing the cells three times with PBST, which contains 0.1% Triton-X100 (Sigma-Aldrich, St. Louis, MO, USA) and is added to PBS, with each wash lasting 5 min. Blocking the cells with a solution of PBST containing 0.5% serum and 0.1% Triton-X100 at room temperature for 1 h. Diluting the primary antibody with blocking buffer according to the instructions or the known ratio before incubating at 4 °C overnight. Washing the cells three times with PBST containing 0.05% TWEEN-20, with each wash lasting 5 min. The secondary antibody was diluted with PBS, usually at a ratio of 1:1000 according to the instructions, and was incubated at room temperature in the dark for 1 h. PBST (0.05% TWEEN-20) was used to wash 3 times for 5 min each time. The cell nuclei was stained with DAPI diluted in PBS according to the instructions, typically at a ratio of 1:5000, and was incubated in the dark at room temperature for 10–15 min. The cells were washed three times with PBST containing 0.05% TWEEN-20, with each wash lasting 5 min.

### 2.8. Statistical Analysis

The Origin and GraphPad Prism 8 statistical software (GraphPad Prism 8) packages were used for the data analysis. The results were averaged from at least three independent experiments and are presented as the means ± the SD. The Student’s *t*-test or one-way analysis of variance (ANOVA) was performed to calculate the statistical significance of the differences. Statistical significance was set at * *p* < 0.05. Semi-quantitative image analysis was performed using Image J software (Image J 1.8.0_172).

## 3. Results and Discussion

### 3.1. Impedance Monitor

**Figure 4:**
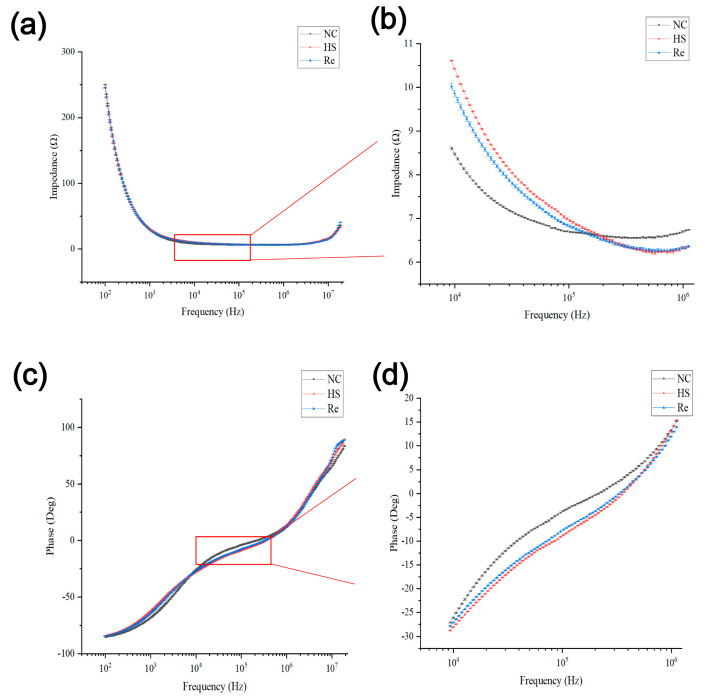
The measurement of the impedance of HUVECs on the biochip. When the frequency was concentrated from 10^2^ to 10^7^ Hz, the amplitude and phase changes are shown in (**a**,**b**). The characterized frequencies ranged between 10^4^ and 10^6^ Hz, as shown in panels (**c**,**d**). NC represents the control group, HS represents the 43 °C heatstroke group, and RE represents the 37 °C 6 h recovery group.

TEER measurement is a rapid, conventional, and non-invasive assay used to assess the epithelial monolayers’ level of integrity and differentiation in in vitro cultures. The electrical impedance across the epithelium or endothelium is related to the formation of robust tight junctions between neighboring cells [22]. As presented in Figure 4, when impedance was measured in the control group and the experimental group, the original impedance spectrum data obtained were plotted according to the measured impedance and phase values at the frequency from 100 Hz to 1 MHz: The amplitude curve in Figure 4a shows a decreasing trend with the increase in frequency. The phase curve obtained in Figure 4c shows a gradual upward trend with the increase in frequency. Amplifying the frequency range from 10^4^ Hz to 10^6^ Hz revealed alterations in the amplitude (Figure 4b) and phase (Figure 4d) curves of the NC (Negative control), HS (Heatstroke), and Re (Recovery) groups.

### 3.2. TEER, Cell-Cell Adhesion Capacitance Monitor

We obtained the TEER and C_I_ values by calculating the impedance spectrum using formulas as follows:

TEER = (a^2^ + b^2^)/a (1)

C_I_ = b/ω (a^2^ + b^2^) (2)

ω = 2πf(3)

where the f represents the frequency measured using an impedance analyzer. Rs represents a constant value in the fitted curve of impedance measurement data, a represents the real part of the impedance measurement data minus Rs, and b represents the imaginary part of the impedance measurement data. The details of the calculation are provided in the Supplementary Materials.

**Figure 5:**
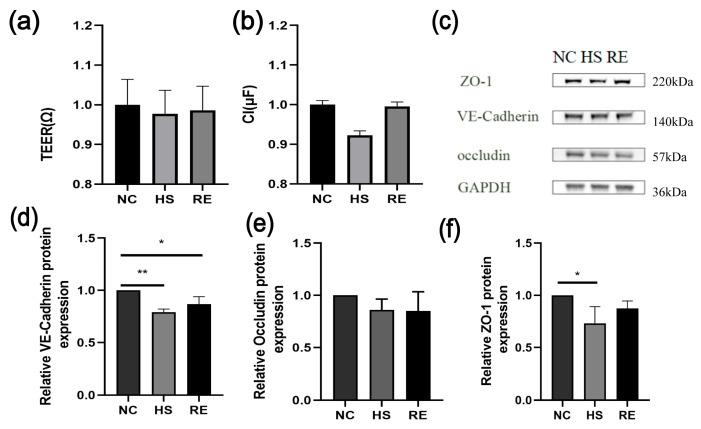
Changes in the TEER and C_I_ values of impedance measurements, and the protein expression of VE-cadherin, occludin, and ZO-1 after heatstroke. NC represents the control group, HS represents the 43 °C heatstroke group, and RE represents the recovery group at 37 °C for 6 h. (**a**,**b**) The TEER and C_I_ values were obtained by the impedance measurement data and the formula. (**c**–**f**) occludin, VE-Cadherin, and ZO-1 protein expression were measured by immunoblotting. (n = 3). The data were quantified by normalization to GAPDH (n = 3). The data are expressed as the means ± the SD. Statistical significance is indicated as * *p* < 0.05, ** *p* < 0.01.

By Equations (1) and (2), the TEER and C_I_ can be calculated. It is interesting to note that the TEER value and C_I_ value of the cells both changed, and both exhibited a downward trend after heat stress (Figure 5a,b). A previous study has shown that the TEER value is related to temperature [13] and the adhesion junctions between cells decreased while the permeability increased [1]. As is shown in Figure 5a,b, our results are consistent with these findings. To further verify whether this conclusion is valid, we collected protein samples from the cells and observed the expression of tight junction proteins. The experimental results showed that the expression of tight junction proteins decreased after heatstroke. This result is consistent with the above conclusions. Therefore, we can deduce that microfluidic impedance measurement can monitor the changes of in cell heatstroke, and this phenomenon may be applied to other disease models. The results are presented in Figure 5a,b. The TEER values of the HUVECs monolayer exposed to heatstroke showed a notable decrease, only to increase again upon recovery. Likewise, a similar trend was demonstrated by the C_I_ values when compared to the NC group. The outcomes demonstrated that the cellular barrier was compromised, leading to an increase in membrane permeability.

To verify whether TEER and C_I_ values were consistent with the biological changes of endothelial cells after heatstroke, we extracted the total protein of the endothelial cells and found that compared with the NC group, the expression of VE-cadherin displays a noticeable reduction in the HS group, and the difference exhibited statistical significance (** *p* < 0.01). Moreover, after recovery, the expression level of VE-cadherin demonstrates an increase with a statistically significant difference (* *p* < 0.05) (Figure 5d). Similarly, ZO-1 expression levels showed a significant reduction after heatstroke treatment compared to the NC group (* *p* < 0.05). Nonetheless, ZO-1 expression levels significantly increased after recovery, exhibiting a statistically significant difference (* *p* < 0.05) (Figure 5e). Occludin expression levels decreased after heatstroke treatment but increased after recovery (Figure 5f).

### 3.3. Immunofluorescence and qPCR Experiment

**Figure 6:**
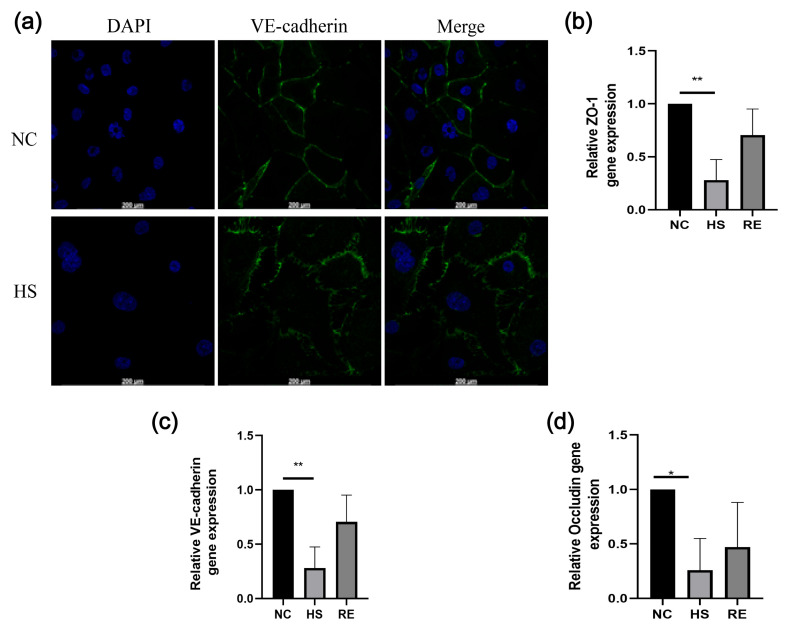
Immunofluorescence Assays and the RNA expression of VE-cadherin after heatstroke. NC represents the control group, HS represents the heatstroke group at 43 °C, and RE represents the recovery group at 37 °C for 6 h. (**a**) presents the alterations in VE-cadherin morphology in HUVEC cells after heatstroke, as contrasted with the control group. (**b**–**d**) present VE-cadherin, occludin, and ZO-1 protein expression were measured by qPCR. The data were quantified by normalization to GADPH (n = 3). The data are expressed as the means ± the SD. Statistical significance is indicated as * *p* < 0.05, ** *p* < 0.01. The scale bar of immunofluorescence map is 200 μm.

To further verify whether the electrical changes were consistent with the biological changes, HUVEC cells were stained, and RNA was extracted after heatstroke. As illustrated in Figure 6a, the VE-cadherin changed from a regular and compact state to a loose state after heatstroke. After heatstroke, VE-cadherin had fewer connections between HUVECs. As presented in Figure 6b–d, after heatstroke, there was a marked decrease in VE-Cadherin mRNA levels (** *p* < 0.01), as well as in the mRNA levels of occludin (* *p* < 0.05) and ZO-1 (** *p* < 0.01). Then, there was an increase in all mRNA levels after recovery. We speculated that it might be due to the migration of occludin from cell membrane to cytoplasm or nucleus during heatstroke, but because of time constraints, we did not verify this phenomenon. In 2017, researchers have found that heatstroke can cause the content of VE-cadherin to decrease in cell membrane and significantly increase in cytoplasm.

## 4. Conclusions

In summary, a biodevice with planar integrated electrodes was devised and tested for culturing and impedance sensing of cells. A new heatstroke model was established to monitor heatstroke in vitro. The electrical properties of heatstroke were obtained by fitting theoretical models to the impedance data. During the process, the value of TEER and C_I_ decreased and that is consistent with the reduction in cell-cell adhesion and cell permeability characterized by proteins (Occludin, VE-Cadherin and ZO-1) and RNA level. Since the changes in occludin protein level, mRNA level and morphology were not obvious after heatstroke. This device can be used to obtain label-free and non-invasive electrical measurements of heatstroke, and it can also be used to investigate other entities, such as the blood brain barrier (BBB). This device can be adapted to monitor dynamic changes in the electrical properties of tissues (organoids) over long periods for biomedical and biological applications.

## Figures and Tables

**Table 1:** Primer names and sequences used in qRT-PCR.

Primers	Primers’ Sequence
GAPDH-forward	GGAGCGAGATCCCTCCAAAAT
GAPDH-reverse	GGCTGTTGTCATACTTCTCATGG
occludin-forward	ACAAGCGGTTTTATCCAGAGTC
occludin-reverse	ACAAGCGGTTTTATCCAGAGTC
VE-Cadherin-forward	GTTCACGCATCGGTTGTTCAA
VE-Cadherin-reverse	CGCTTCCACCACGATCTCATA
ZO-1-forward	ATGTTGCTCTACACCCTGACC
ZO-1-reverse	CCAGCACACACATAGATCCAGT

**Table 2:** qRT-PCR reaction system for each well.

Reagent	Volume (μL)
cDNA	1
2.5 μM Primer Forward	0.4
2.5 μM Primer Reverse	0.4
Nuclease-Free Water	3.2
2×SYBR Green	5
Total volume	10

## Data Availability

The data that support the findings of this study are available from the corresponding author upon reasonable request.

## Supplementary Materials

The following supporting information can be downloaded at: https://www.mdpi.com/article/10.3390/bios13070686/s1.
