# Supplementary material for: Non-Invasive and Label-Free On-Chip Impedance Monitoring of Heatstroke
Source: Biosensors (Basel). 2023 Jun 27;13(7):686. doi: 10.3390/bios13070686 (PMC10377158; doi:10.3390/bios13070686)
Supplement: Supplementary file 1 [file biosensors-13-00686-s001.zip › biosensors-2314594-supplementary.pdf]

Supporting Information

Non-Invasive and Label-Free On-Chip Impedance Monitoring of Heatstroke

Yueli Zhao [1,2,3], Weihua Fan [2], Anwei Liu [3], Shihua Pan [2,4], Chongxiao Xu [3,5], Hailun Peng [3,5], Bingling Yin [1,3], Xiaodong Wang [6], Jianhua Dong [2] and Zhiguo Pan [1,3,5,*]

[1] Guangzhou University of Chinese Medicine, Guangzhou 510006, China; 20201111187@stu.gzucm.edu.cn (Y.Z.); 20221111561@stu.gzucm.edu.cn (B.Y.)
[2] Guangzhou Institute of Biomedicine and Health, Chinese Academy of Sciences, Guangzhou 510530, China; fan_weihua@gibh.ac.cn (W.F.); 2020219451@stu.gzhmu.edu.cn (S.P.); dong_jianhua@gibh.ac.cn (J.D.)
[3] Department of Intensive Care Unit, General Hospital of Southern Theatre Command of PLA, Guangzhou 510000, China; anweiliu2014@163.com (A.L.); fanteny@tom.com (C.X.); 17825846221@163.com (H.P.)
[4] Department of Biochemistry and Molecular Biology, GMU-GIBH Joint School of Life Sciences, Guangzhou Medical University, Guangzhou 511436, China
[5] Southern Medical University, Guangzhou 510000, China
[6] Engineering Research Center for Semiconductor Integrated Technology, Institute of Semiconductors, Chinese Academy of Sciences, Beijing 100083, China; xdwang@semi.ac.cn
* Correspondence: 13060686138@126.com; Tel.: +86-130-6068-6138

Calculating values of TEER and C_I

As described in figure 3 in the context, the values of TEER and C_I can be calculated as following:

For no cells (Figure 3b) condition, the whole impedance can be seen as:

$$Z_0 = R_s - j\frac{1}{\omega C_{dl}} = Z_{0re} + jZ_{0im} \qquad \text{Eq (S1)}$$

Where ω represents angle frequency, and R_s represents the resistance of medium.

For monolayer HUVECs (Figure 3c) condition, the impedance is described as:

$$Z = R_s + \frac{1}{\frac{1}{TEER} + j\omega C_I}$$

$$= R_s + \frac{TEER}{1 + j\omega C_I \cdot TEER}$$

$$= R_s + \frac{TEER}{1 + \omega^2 {C_I}^2 \cdot TEER^2} - j\frac{\omega C_I \cdot TEER^2}{1 + \omega^2 {C_I}^2 \cdot TEER^2}$$

$$= Z_{re} + jZ_{im} \qquad \text{Eq (S2)}$$

Where the C_I is capacitance of cell-cell adhesion, and Z_{0re}, Z_{0im}, Z_{re} and Z_{im} are real and imagery of no cell and monolayer HUVECs respectively.

By combining eq(S1) and (S2), and supposing:

$$a = Z_{re} - Z_{0re} = \frac{TEER}{1+\omega^2 {C_I}^2 \cdot TEER^2} \qquad \text{Eq (S3)}$$

$$b = Z_{im} = \frac{\omega C_I \cdot TEER^2}{1+\omega^2 {C_I}^2 \cdot TEER^2} \qquad \text{Eq (S4)}$$

Combining eq (S3) and (S4), the values of TEER and C_I are calculated as:

$$TEER = \frac{a^2 + b^2}{a}$$

$$C_I = \frac{b}{\omega(a^2 + b^2)}$$
